# Consumption Patterns of Processed Foods in Singapore—A Cross-Sectional Study

**DOI:** 10.3390/foods11182782

**Published:** 2022-09-09

**Authors:** Patrick Gan, Jun Cheng Er, Kenneth Chow, Benjamin Er, Joanne Sheot Harn Chan, Angela Li, Kyaw Thu Aung

**Affiliations:** 1 National Centre for Food Science, Singapore Food Agency, 7 International Business Park, Singapore 609919, Singapore; 2 School of Biological Sciences, Nanyang Technological University, 60 Nanyang Dr, Singapore 637551, Singapore

**Keywords:** processed foods, consumption pattern, sociodemographic factors, food frequency questionnaire

## Abstract

The consumption of processed foods is increasingly widespread and could have an impact on diet quality and health. Understanding the factors influencing people’s eating habits is useful for assessing such impact. There are limited data on the consumption patterns of processed foods and associated factors influencing the dietary patterns in Singapore. This cross-sectional study based on a food frequency questionnaire aimed to examine how the consumption of processed foods among 2079 Singapore residents aged 18 to 89 years varies with sociodemographic factors. The analysis of the consumption by processed food groups showed that the studied factors, i.e., age, gender, ethnicity, housing and health status, all contributed to differences in processed food consumption to varying extents, with ethnicity being the key factor driving the variation. Such differences were also confirmed to a limited degree by determining another measure of consumption, i.e., a processed food variety score. The findings in this study could inform further work in relation to dietary risks.

## 1. Introduction

Food processing has improved the palatability and shelf-life of foods; however, food processing could lead to undesirable effects, including the loss of nutrients from heat treatment and the formation of toxic compounds, such as N-nitroso-compounds in processed meat and acrylamide in potato chips [1]. Studies have also suggested the association of increased processed food consumption with an increase of obesity and chronic non-communicable diseases [2,3,4,5,6,7].

Processed food items refer to foods which have undergone at least some processing and thus include moderately and highly processed foods [8]. With the modern food technologies, the range and volume of processed foods have increased. This has led to an expansion in the consumption of processed foods, especially in high- and middle-income countries [9]. Given the significant concerns linked to the growing intake of processed foods, there is a need to understand the consumption patterns and associated factors that influence the consumption of processed foods. Sociodemographic differences have been reported in studies on food consumption [10,11,12]. Various factors such as ethnicity [13], gender [10], age [14], socioeconomic status [10,15] and health status [16] showed an influence on food intake patterns. The examination of consumption patterns and associated factors would help understand the variation of the eating habits with these factors and ultimately would be useful to develop an evidence-based approach to food safety risk assessments or dietary intervention measures [13].

Various approaches are available to investigate an individual’s food consumption, including 24 h recalls, food records and food frequency questionnaires (FFQ) [17], with the choice of the methodology dependent on the research objective. While survey instruments such as 24 h recalls and food records require low memory efforts by the respondents, the time frame of the consumption that can be studied tends to be short. In contrast, FFQ are more appropriate for a query on diet over a longer time frame. Moreover, the food items surveyed in the FFQ can focus on specific components of interest in the diet.

In Singapore, the demographic profile of processed foods consumption has not been widely studied. There was a previous report on fast food consumption among adult Singapore residents; however, it was limited to five fast food items in the questionnaire [18]. This paper aimed to examine the variation of consumption of a range of processed foods in Singapore by socio-demographic factors based on the FFQ design. Findings from this study on the sociodemographic characteristics of processed foods consumption will provide information for further studies in relation to dietary risks.

## 2. Materials and Methods

### 2.1. Study Design

This study uses a cross-sectional design with a random sample representative of Singapore residents (Singapore citizens and permanent residents) aged 18 to 89 years, consisting of 2079 respondents stratified by gender, age and ethnicity, as shown in Table 1. The survey adopted a three-step stratified random sampling design:

1. The primary sampling unit (PSU) was the residential block (or street for landed properties). A fixed number of household addresses, stratified by geographic zones and housing types, was randomly selected, taking into consideration the five geographical zones of Singapore;
2. The secondary sampling unit was the residential household unit. Interviewers started from the biggest unit number from the street or the smallest unit number from the block based on the PSU. Every third unit was approached until the street or block was covered;
3. The tertiary sampling unit was the household member. The selection of the household member followed the last birthday methodology, with only one member selected from each household for the interview.

**Table 1:** Demographic distribution of the respondents in the food consumption survey.

	% Total Respondents (*n* = 2079)	*n*
**Gender**		
Female	51.9	1080
Male	48.1	999
**Ethnic group**		
Chinese	73.4	1525
Malay	13.4	278
Indian	9.3	194
Others	3.9	82
**Age group (years)**		
18–39	40.4	840
40–59	34.6	720
60–89	25.0	519
**Housing type**		
HDB 1–3 Room ^1^	19.4	404
HDB 4–5 Room	68.2	1417
Condominiums/Private Apartments	7.0	146
Terrace/Semi-Detached/Bungalow	5.4	112
**Self-reported health conditions**		
Has at least one health condition	26.2	545
No health condition	73.8	1534

^1^ HDB: Housing Development Board (refers to public housing).

The respondents answered a food frequency questionnaire (FFQ) in face-to-face interviews from October to December 2020, conducted by interviewers from a third-party research agency. Informed verbal consent was obtained from the participants prior to the interview. The respondents were asked the frequency and number of servings of a list of 223 processed food items they consumed during a span of the previous six months. The list covered those less commonly eaten processed foods identified based on food import statistics. The questionnaire also included questions on socio-demographics, such as age, ethnicity, gender and housing type. As an indicator of the socioeconomic status (SES) [19,20], housing type is categorised into (1) HDB apartments 1–3 room, (2) HDB apartments 4–5 room, (3) condominiums and private apartments, and (4) terrace houses, semi-detached houses and bungalows. HDB apartments refer to public housing provided by the Housing and Developing Board in Singapore. Self-reported body weight and existing health conditions such as diabetes, high blood pressure and high cholesterol were also recorded.

### 2.2. Data Analysis

Processed foods in this study refer to foods involving at least some processing, including moderately and highly processed foods as classified in the IARC system [8]. The list of processed food items was sorted into 12 food groups, adapted from the food categorisation system for the Codex Alimentarius General Standard for Food Additives [21], with the detailed breakdown provided in the Supplementary Information:

- Beverages, excluding dairy products;
- Cereals and cereal products;
- Confectionery and sweeteners;
- Dairy products;
- Fish and seafood;
- Fruits;
- Meat and meat products;
- Nuts and seeds;
- Sauces and condiments;
- Seaweed and fungi;
- Soups;
- Vegetables.

The categorisation in this study was based on a food import classification, with an expected similarity of food availability through imports to consumption patterns, as Singapore relies heavily on imports for food.

Descriptive statistics are presented for mean and median daily food consumption per food group among consumers by gender, age, ethnicity and existing health conditions. The diversity of processed food consumption was measured using the Processed Food Variety Score (PFVS), defined as the number of different processed food groups consumed by each respondent [22], using a reference period of the past six months prior to the interview. The differences between means were evaluated by one-way analysis of variance (ANOVA) and independent *t*-test, with significance of α ≤ 0.05. Following ANOVA, post hoc analyses using Tukey’s HSD tests were conducted to compare the means between any two groups.

### 2.3. Ethical Considerations

The study was approved by the National Centre for Food Science’s Project Review Committee (Project ID RAP20.1), Singapore Food Agency. Verbal informed consents were obtained from all respondents involved in this study. Individual respondents were delineated by an identifier to maintain respondents’ anonymity.

## 3. Results

A total of 2079 respondents participated in the food consumption survey, with the demographic distribution described in Table 1.

As shown in Table 2, the proportion of the sample population consuming each processed food category ranged from 35.6% for “soups” to 97.6% for processed “cereals and cereal products”. Ten out of the twelve processed food categories were consumed by least two-thirds of the sample population, except for “soups” and “sauces and condiments” which were consumed by 35.6% and 56.3% of the sample population, respectively.

**Figure 1:**
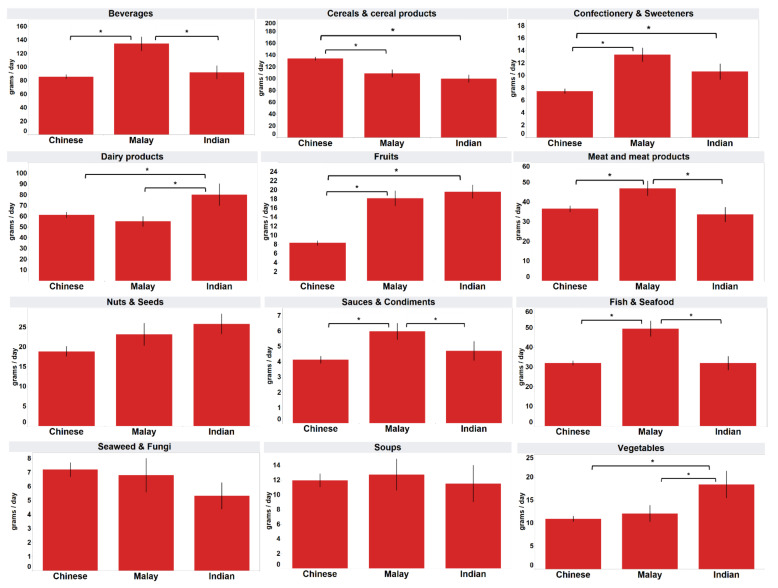
Mean daily intake of the consumers, considering the top three ethnic groups. * Mean values were significantly different as determined by ANOVA with Tukey’s HSD test for multiple comparisons (*p* < 0.05).

Ethnic differences in processed food category consumption by the three main ethnic groups are shown in Figure 1. The Chinese respondents consumed significantly more processed “cereals and cereal products” but significantly less “confectionery and sweeteners” and processed “fruits” than the Malay and Indian respondents (*p* < 0.05). In contrast, the Malay respondents consumed significantly more processed “fish and seafood”, “meat and meat products”, “sauces and condiments” and “beverages” than the Chinese and Indians (*p* < 0.05). On the other hand, the mean consumptions of “dairy products” (mean 80.3 g) and processed “vegetables” (mean 18.4 g) by the Indian respondents were significantly higher than those of the Chinese (mean 61.4 g, 10.9 g) and Malay (mean 55.3 g, 12.1 g) respondents (*p* < 0.05).

**Figure 2:**
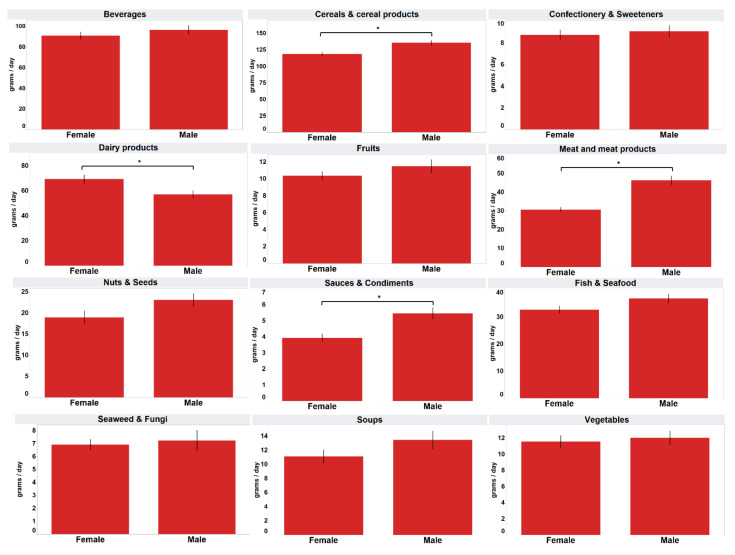
Mean daily intake of the consumers analysed by gender. * Mean values were significantly different as determined by independent *t*-tests (*p* < 0.05).

Figure 2 shows the mean daily intake of processed food categories by gender. Males consumed significantly more processed “cereals and cereal products” (mean 135.4 g), “meat and meat products” (mean 46.4 g) and “sauces and condiments” (mean 5.5 g) than females (mean 118.4 g, 30.7 g, 3.9 g) (*p* < 0.05). However, the mean consumption of “dairy products” was significantly greater for females (mean 69.3 g) than for males (mean 57.1 g) (*p* < 0.05).

**Figure 3:**
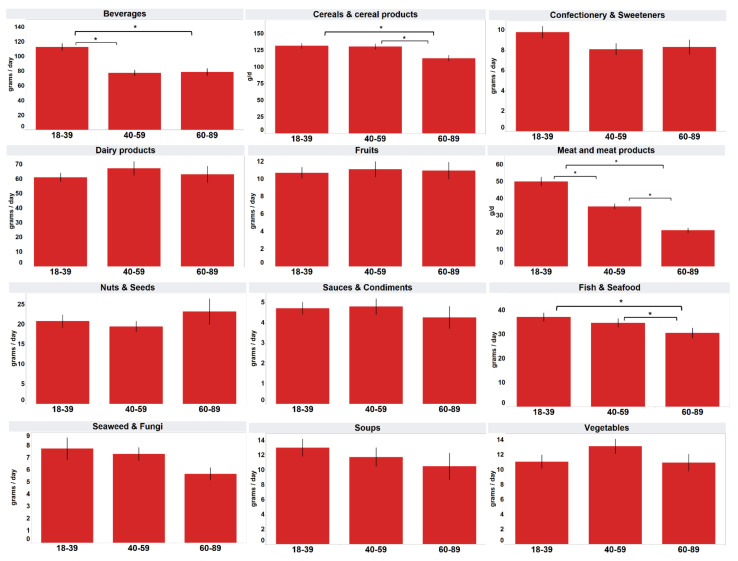
Mean daily intake of consumers analysed by age group. * Mean values were significantly different as determined by ANOVA with Tukey’s HSD test for multiple comparisons (*p* < 0.05).

The mean consumption of processed “meat and meat products” among the three age groups was significantly different (*p* < 0.05), with decreasing consumption as age increased (Figure 3). Younger age groups including 18-to-39-year-old and 40-to-59-year-old respondents consumed significantly more “cereals and cereal products” (mean 131.4 g, 130.4 g) and “fish and seafood” (mean 37.2 g, 34.8 g) than the oldest age group comprising respondent of 60 to 89 years of age (mean 112.9 g, 30.5 g) (*p* < 0.05). The youngest age group of 18-to-39-year-old participants (mean 112.6 g) consumed significantly more “beverages” than the 40-to-59-year-old (mean 77.3 g) and 60-to-89-year-old age groups (mean 78.6 g) (*p* < 0.05).

**Figure 4:**
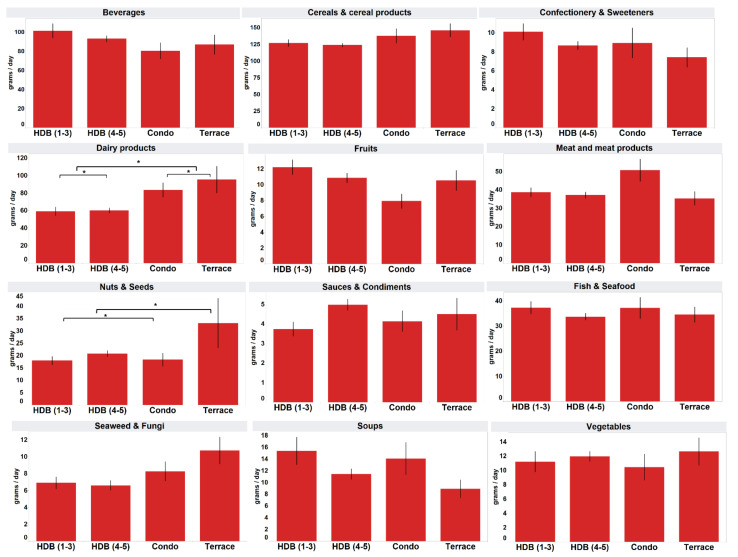
Mean daily intake of consumers analysed by housing type. * Mean values were significantly different as determined by ANOVA with Tukey’s HSD test for multiple comparisons (*p* < 0.05). HDB refers to public housing, with the number of rooms in the apartment reported in brackets.

The respondents residing in Terrace/Semi-Detached/Bungalow and Condominiums/Private Apartments consumed significantly more “dairy products” (mean 95.4 g, 83.6 g) than those living in HDB 1–3 Room (mean 59.2 g) and HDB 4–5 Room (mean 60.2 g) (*p* < 0.05) (Figure 4). The residents of Terrace/Semi-Detached/Bungalow (mean 33.2 g) also consumed significantly more processed “nuts and seeds” than the residents of HDB 1–3 Room (mean 18.0 g), HDB 4–5 Room (mean 20.8 g) and Condominiums/Private Apartments (mean 18.4 g) (*p* < 0.05).

**Figure 5:**
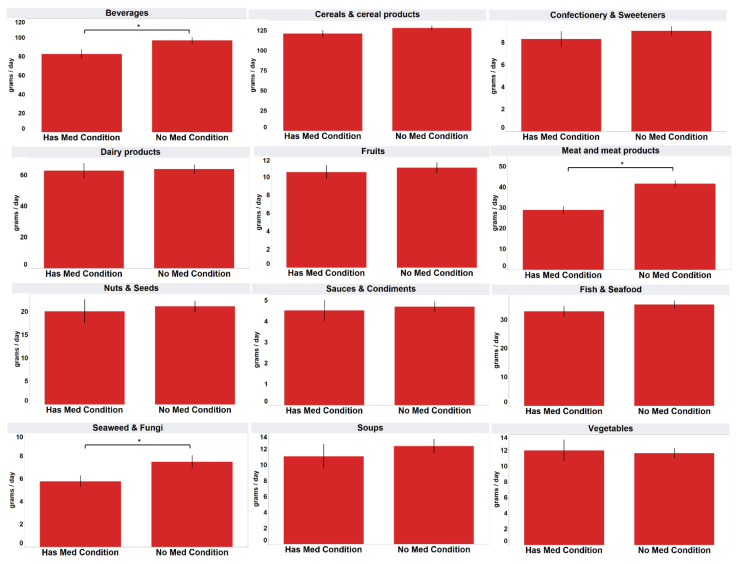
Mean daily intake of consumers analysed by self-reported health conditions. * Mean values were significantly different as determined by independent *t*-test (*p* < 0.05). ‘Has Med Condition’ refers to having at least one health condition such as diabetes, high blood pressure or high cholesterol.

The respondents without existing health conditions consumed significantly more processed “meat and meat products” (mean 41.7 g), “seaweed and fungi” (mean 7.5 g) and “beverages” (mean 97.0 g) than those with at least one self-reported existing health condition (mean 29.0 g, 5.8 g, 82.6 g) (*p* < 0.05) (Figure 5).

The mean Processed Food Variety Score (PFVS) of the sample population was 9.03 (Table 3). Significant differences in the mean PFVS were found for all sociodemographic factors. The greatest diversity, with significantly higher mean PFVS (*p* < 0.05), was observed for males, respondents of Chinese ethnicity, respondents who were 18 to 39 years old, respondents residing in terrace/semi-detached/bungalow and respondents with no self-reported health conditions.

Table 4 shows the mean daily intake analysed by cross-tabulation of gender and age group. The mean consumption of “beverages” and processed “meat and meat products” among the three age groups was significantly different (*p* < 0.05) for both males and females, with the same trend as that in Figure 3 (age groups alone). For processed “cereals and cereal products” and “dairy products”, only males of different age groups showed a significant difference (*p* < 0.05) in mean consumption, while for processed “fish and sea-food”, only females of different age groups showed a significant difference (*p* < 0.05) in mean consumption.

As shown in Table 5, significant ethnic differences (*p* < 0.05) in relation to the mean consumption of “beverages”, “confectionery and sweeteners” and processed “fruits” were observed for both males and females, with a similar trend as that in Figure 1 (ethnic groups alone). For processed “cereals and cereal products” and “nuts and seeds”, only males of different ethnic groups showed a significant difference (*p* < 0.05) in mean consumption. In contrast, the mean consumptions of “dairy products”, processed “fish and seafood”, “meat and meat products”, “vegetables” and “sauces and condiments” were significantly different (*p* < 0.05) among the three ethnic groups only for females.

## 4. Discussion

The findings from this cross-sectional study showed that the majority (10 out of 12) of the processed food groups were consumed by more than two-thirds of the sample population for at least once in the six months preceding the survey. The consumption of certain processed food groups varies with sociodemographic factors. In particular, the largest variations were observed for ethnic groups, which showed differences for 9 out of the 12 food groups. A study found that the dietary practices among Chinese, Malays and Indians in Singapore have significant differences [23]. Such dietary variation also extends to processed foods consumption, as shown in this study. For instance, the higher consumption of processed “vegetables” by Indians than by other ethnic groups in this study could be due to the prevalence of Hinduism in this ethnic group [23]. The increased consumption of certain processed food groups such as “meat and meat products” and “confectionery and sweeteners” by Malays is similar to that reported for Malays in the Malaysian population, who also were shown to consume more of these food groups relative to Chinese and Indians [24], although the reason for these preferences is unclear.

Among the three ethnic groups that comprised 96.1% of the sample population, the Malay respondents reported a significantly higher mean consumption of six of the processed food groups, whereas the Chinese and Indian respondents showed a significantly higher mean consumption of one and four processed food groups, respectively. The observed pattern in the food consumption habits from these surveys could be due to cultural preferences [23,25]. For instance, in a previous study on the consumption of fast food in Singapore, the proportion of consumers was the highest among the Malay residents compared to the Chinese and Indians [18].

There was relatively less variation of processed food group mean consumption in terms of gender and age, with differences in mean consumption found for four processed food groups each. For those food groups with differences, a higher mean consumption tended to be observed among males and younger respondents. Other studies also reported similar findings of men and younger adults consuming more processed foods in UK [6], Canada [5], Australia [26] and South Korea [27]. The increased consumption of processed foods could be due to the convenience [28] and popularity [9] of such foods, as well as the relative lack of time and food preparation skills [29].

Only two processed food groups, “dairy products” and “nuts and seeds”, showed significant differences in mean consumption by housing types, a criterion used as an indicator of the socioeconomic status (SES) [19,20]. As Singapore has a high home ownership rate of 87.9% and a low unemployment rate of 3.0% [30], housing information serves as a relevant marker of SES [19]. Those residing in private housing, indicative of a higher SES, consumed more of these two processed food groups. This is similar to previous findings indicating that a higher income was associated with a greater consumption of dairy products in China and Brazil [31,32], where dairy products intake is not so prevalent in the diet. While peanuts are commonly available [33], more expensive tree nuts are considered a relative luxury food whose consumption was positively related to the purchasing demand by higher income [34].

The respondents reporting existing health conditions of diabetes, high blood pressure and high cholesterol in this study consumed similar mean amounts of 9 out of the 12 processed food groups and significantly lower amounts of processed “meat and meat products”, “seaweed and fungi” and “beverages” compared to respondents without existing health conditions. The lower mean consumption of certain food groups by the respondents with existing health conditions could be due to awareness of their health status, leading them to avoid certain types of food [35]. On the other hand, a preventive approach should be taken for those without existing health conditions to increase consumer education and awareness regarding the dietary habits.

There are studies showing that the intake of certain processed foods, in particular those based on industrial formulations that typically contain many ingredients including additives [36], was associated with a higher prevalence of obesity, diabetes and hypertension [3,5]. However, other aspects of the diet such as the consumption of fresh vegetables and fruits are inversely associated with chronic diseases such as cardiovascular disease [37,38]. More research is needed to identify the impact of other diet components in relation to the processed foods and also the influence of other risk factors such as lifestyle.

The processed food variety score (PFVS) measures the number of different processed food groups consumed and thus the diversity of processed food consumption [22,39,40]. The mean score of 9.03 for the sample population out of a maximum of 12 indicated a relatively wide range of processed food groups consumed, consistent with the trend of a growing processed food consumption worldwide [9,41]. Significant differences in PFVS were found for each of the sociodemographic factors studied, reflecting the observed variations in consumption of processed food groups. The PFVS could act as a proxy for the estimation of the consumption amounts in identifying variables with significant differences, given that previous reports suggested that the variety scores are positively associated with food consumption [42,43]. Such a qualitative approach could be useful for monitoring the consumption amounts where resources are limited to conduct a full study at the food item level. Although the PFVS could be used as a predictor of the consumption levels to a limited degree, the extent of the variation for various processed food groups can only be assessed by conducting a more detailed survey evaluating the quantitative food intake.

In the cross-tabulation of gender and age groups, the pattern of the presence or absence of a significant difference in the mean consumption among the age groups was largely the same as that obtained with the analysis for age groups alone, when gender was not considered. However, the difference in mean consumption of “cereals and cereal products” among different age groups was driven by males, while the difference in mean consumption of “fish and seafood” among different age groups was driven by females. Both categories showed significant differences when considering gender alone.

When gender and ethnic groups were cross-tabulated, significant differences in the mean consumption of processed food groups among ethnic groups were observed for only males (two food groups), only females (five food groups) or both genders (three food groups). This suggests that the observation of significant differences when analysing ethnic groups alone could be driven by one of the genders for certain processed food groups.

This study has some limitations. First, the cross-sectional design of this study limited the inference of causality [44], since the findings provide only a snapshot of the association of the studied factors with the food consumption patterns. Similarly, the influence of the COVID-19 pandemic on the reported intake could not be assessed. Some studies have reported that the COVID-19 pandemic has affected food consumption in certain populations [45,46], while other studies reported that food consumption remained largely the same [47,48]. As this study was conducted during the COVID-19 pandemic, an influence of the pandemic on the reported intake cannot be precluded. Second, as there are several ways to classify processed foods [49], it is possible that other food classifications could have been applicable to assess the consumption patterns. These food classifications [8,50,51,52,53] are based on the degree of processing, each using different criteria. The various classifications could vary in the categorisation of processed foods [54], even the increasingly popular NOVA classification system could present possible inconsistencies [55,56]. As a standardised way to classify processed foods is still lacking, the categorisation in this study was based on the type of processed foods rather than on the degree of processing. Third, the existing health conditions reported by the respondents were not differentiated by whether they were hereditary or acquired; thus, any association between the onset of the conditions and processed foods consumption is not known. As this was a cross-sectional study, the observed consumption pattern was limited to a short period and could not necessarily influence the health conditions of the respondents. Fourth, this study was based on the food frequency questionnaire which requires the respondents to recall their food consumption over a period of time. This could result in some over- or under-reporting due to recall bias [57]. Nevertheless, our findings contribute to the limited literature on the influence of sociodemographic factors on processed food groups consumption in Singapore and provide a basis for future comparisons of the local consumption status over a longer time period, noting the limitations of the study as discussed.

In conclusion, gender, age, ethnic group, housing and health status, all contributed to differences, to varying extents, in the consumption of certain processed food groups in Singapore, with ethnicity being the key factor in driving the variations. The differences arising from these sociodemographic factors were also reflected in the processed food variety score. These results can be useful for an evidence-based approach to food safety risk assessment or dietary intervention measures. In addition, these findings can facilitate further research on dietary patterns. As this is the first cross-sectional study on the consumption of processed foods in Singapore, future iterations of consumption surveys are recommended to evaluate the trends in the consumption patterns of processed foods.

## Figures and Tables

**Table 2:** Proportion of consumers of each food category.

Processed Food Category	% Consumers
Cereals and cereal products	97.6
Fish and seafood	89.0
Meat and meat products	88.8
Beverages, excluding dairy products	83.7
Dairy products	80.5
Fruits	80.0
Nuts and seeds	77.3
Confectionery and sweeteners	76.9
Seaweed and fungi	70.3
Vegetables	67.7
Sauces and condiments	56.3
Soups	35.6

**Table 3:** Processed food variety score by sociodemographic factors.

		Processed Food Variety Score			
	*n*	Mean	SE	Minimum	Maximum
Sample Population		9.03	0.05	1	12
**Gender**					
Female	1080	8.81 *	0.07	2	12
Male	999	9.24	0.06	1	12
**Ethnic group**					
Chinese	1525	9.14 ^a^	0.05	1	12
Malay	278	8.82 ^a,b^	0.13	3	12
Indian	194	8.43 ^b^	0.16	2	12
**Age group (years)**					
18–39	840	9.50 ^a^	0.07	3	12
40–59	720	9.06 ^b^	0.08	1	12
60–89	519	8.25 ^c^	0.10	1	12
**Housing type**					
HDB 1–3 Room	404	8.68 ^a^	0.11	1	12
HDB 4–5 Room	1417	9.05 ^b^	0.06	2	12
Condominiums/Private Apartments	146	9.08 ^a,b^	0.20	2	12
Terrace/Semi-Detached/Bungalow	112	9.96 ^c^	0.17	3	12
**Self-reported health conditions**					
Yes	545	8.42 *	0.09	1	12
No	1534	9.26	0.05	1	12

* Mean values between males and females were significantly different as determined by independent *t*-tests (*p* < 0.05). ^a,b,c^ Mean values with different superscript letters were significantly different as determined by ANOVA with Tukey’s HSD test for multiple comparisons (*p* < 0.05).

**Table 4:** Mean daily intake of consumers analysed by gender and age group.

Processed Food Group	Male			Female		
	18–39 years (*n* = 400)	40–59 years (*n* = 333)	60–89 years (*n* = 266)	18–39 years (*n* = 440)	40–59 years (*n* = 387)	60–89 years (*n* = 253)
Beverages, excluding dairy products	119.7 ^a^	76.9 ^b^	77.7 ^b^	106.3 ^a^	77.7 ^b^	79.6 ^b^
Cereals and cereal products	138.2 ^a^	145.8 ^a^	117.4 ^b^	125.3	117.2	108.4
Confectionery and sweeteners	10.3	7.6	8.8	9.4	8.5	7.9
Dairy products	58.0 ^a^	64.6 ^a^	43.2 ^b^	63.9	69.3	80.8
Fish and seafood	37.9	37.7	33.9	36.4 ^a^	32.2 ^ab^	27.0 ^b^
Fruits	10.5	12.2	12.1	10.9	10.2	9.8
Meat and meat products	61.0 ^a^	43.5 ^b^	24.6 ^c^	39.4 ^a^	28.4 ^b^	17.8 ^c^
Nuts and seeds	25.0	21.9	21.7	17.1	17.5	24.8
Sauces and condiments	5.6	5.7	4.4	3.9	4.0	4.1
Seaweed and fungi	8.5	6.8	5.8	7.0	7.7	5.6
Soups	12.5	15.1	13.1	13.4	9.1	8.5
Vegetables	11.3	12.6	12.1	10.9	13.5	9.7

^a,b,c^: Mean values with different superscript letters between age groups for males and females separately were significantly different as determined by ANOVA with Tukey’s HSD test for multiple comparisons (*p* < 0.05). Standard errors (not shown) are available upon request.

**Table 5:** Mean daily intake of consumers analysed by gender and ethnic group.

Processed Food Group	Male			Female		
	Chinese (*n* = 749)	Malay (*n* = 119)	Indian (*n* = 86)	Chinese (*n* = 776)	Malay (*n* = 159)	Indian (*n* = 108)
Beverages, excluding dairy products	90.1 ^a^	135.8 ^b^	104.3 ^ab^	81.6 ^a^	133.2 ^b^	82.3 ^a^
Cereals and cereal products	145.7 ^a^	103.3 ^b^	101.1 ^b^	122.3	112.7	99.1
Confectionery and sweeteners	7.9 ^a^	12.8 ^b^	11.6 ^ab^	7.2 ^a^	13.7 ^b^	9.8 ^ab^
Dairy products	55.0	53.3	62.5	66.9 ^ab^	56.6 ^a^	93.7 ^b^
Fish and seafood	36.0	47.2	29.1	28.2 ^a^	51.1 ^b^	34.3 ^a^
Fruits	9.0 ^a^	18.2 ^b^	22.0 ^b^	7.7 ^a^	18.1 ^b^	17.7 ^b^
Meat and meat products	46.1	53.5	35.8	27.0 ^a^	42.5 ^b^	31.7 ^ab^
Nuts and seeds	19.6 ^a^	29.2 ^ab^	31.9 ^b^	18.2	19.2	21.2
Sauces and condiments	5.0	6.6	5.7	3.3 ^a^	5.5 ^b^	4.2 ^ab^
Seaweed and fungi	7.2	8.7	5.4	7.2	5.6	5.3
Soups	13.0	16.6	14.8	11.1	10.4	9.5
Vegetables	11.5	13.2	15.5	10.5 ^a^	11.4 ^a^	20.6 ^b^

^a,b^: Mean values with different superscript letters between age groups for males and females separately were significantly different as determined by ANOVA with Tukey’s HSD test for multiple comparisons (*p* < 0.05). Standard errors (not shown) are available upon request.

## Data Availability

The data presented in this study are available on request from the corresponding author.

## Supplementary Materials

The following supporting information can be downloaded at: https://www.mdpi.com/article/10.3390/foods11182782/s1, Table S1: Food items in the processed food groups.

## References

[1] Van Boekel M., Fogliano V., Pellegrini N., Stanton C., Scholz G., Lalljie S., Somoza V., Knorr D., Jasti P.R., Eisenbrand G. (2010). A review on the beneficial aspects of food processing. Mol. Nutr. Food Res..

[2] World Health Organization (2003). Diet, Nutrition, and the Prevention of Chronic Diseases: Report of a Joint WHO/FAO Expert Consultation.

[3] Lawrence M.A., Baker P.I. (2019). Ultra-processed food and adverse health outcomes. Brit. Med. J..

[4] Martínez Steele E., Popkin B.M., Swinburn B., Monteiro C.A. (2017). The share of ultra-processed foods and the overall nutritional quality of diets in the US: Evidence from a nationally representative cross-sectional study. Popul. Health Metr..

[5] Moubarac J.C., Batal M., Louzada M.L., Steele E.M., Monteiro C.A. (2017). Consumption of ultra-processed foods predicts diet quality in Canada. Appetite.

[6] Rauber F., Steele E.M., Louzada M.L.D.C., Millett C., Monteiro C.A., Levy R.B. (2020). Ultra-processed food consumption and indicators of obesity in the United Kingdom population (2008–2016). PLoS ONE.

[7] Nardocci M., Polsky J.Y., Moubarac J.C. (2021). Consumption of ultra-processed foods is associated with obesity, diabetes and hypertension in Canadian adults. Can. J. Public Health.

[8] Slimani N., Deharveng G., Southgate D.A.T., Biessy C., Chajes V., Van Bakel M.M.E., Boutron-Ruault M.C., McTaggart A., Grioni S., Verkaik-Kloosterman J. (2009). Contribution of highly industrially processed foods to the nutrient intakes and patterns of middle-aged populations in the European Prospective Investigation into Cancer and Nutrition study. Eur. J. Clin. Nutr..

[9] Monteiro C.A., Moubarac J.C., Cannon G., Ng S.W., Popkin B. (2013). Ultra-processed products are becoming dominant in the global food system. Obes. Rev..

[10] Deshmukh-Taskar P., Nicklas T.A., Yang S.J., Berenson G.S. (2007). Does food group consumption vary by differences in socioeconomic, demographic, and lifestyle factors in young adults? The Bogalusa Heart Study. J. Am. Diet. Assoc..

[11] Eicher-Miller H.A., Fulgoni III V.L., Keast D.R. (2015). Energy and nutrient intakes from processed foods differ by sex, income status, and race/ethnicity of US adults. J. Acad. Nutr. Diet..

[12] Hiza H.A., Casavale K.O., Guenther P.M., Davis C.A. (2013). Diet quality of Americans differs by age, sex, race/ethnicity, income, and education level. J. Acad. Nutr. Diet..

[13] Barkoukis H. (2007). Importance of understanding food consumption patterns. J. Am. Diet. Assoc..

[14] Westenhoefer J. (2005). Age and gender dependent profile of food choice. Diet Diversification and Health Promotion.

[15] Hare-Bruun H., Togo P., Andersen L.B., Heitmann B.L. (2011). Adult food intake patterns are related to adult and childhood socioeconomic status. J. Nutr..

[16] Trondsen T., Braaten T., Lund E., Eggen A.E. (2004). Health and seafood consumption patterns among women aged 45–69 years. A Norwegian seafood consumption study. Food Qual. Prefer..

[17] World Health Organization (WHO) (2009). Dietary Exposure Assessment of Chemicals in Food. Principles and Methods for the Risk Assessment of Chemicals in Food, Environmental Health Criteria 240.

[18] Whitton C., Ma Y., Bastian A.C., Chan M.F., Chew L. (2014). Fast-food consumers in Singapore: Demographic profile, diet quality and weight status. Public Health Nutr..

[19] Chan C.Q.H., Lee K.H., Low L.L. (2018). A systematic review of health status, health seeking behaviour and healthcare utilisation of low socioeconomic status populations in urban Singapore. Int. J. Equity Health.

[20] Sabanayagam C., Shankar A., Wong T.Y., Saw S.M., Foster P.J. (2007). Socioeconomic status and overweight/obesity in an adult Chinese population in Singapore. J. Epidemiol..

[21] World Health Organization (2011). Codex Alimentarius: General Standard for Food Additives.

[22] Drewnowski A., Renderson S.A., Driscoll A., Rolls B.J. (1997). The Dietary Variety Score: Assessing diet quality in healthy young and older adults. J. Am. Diet. Assoc..

[23] Ng R.Y.X., Wong Y.S., Yeo J.Y., Koh C.L.Z., Wilson C., Gan S.K.E. (2018). The associations between dietary practices and dietary quality, biological health indicators, perceived stress, religiosity, culture, and gender in multicultural Singapore. J. Ethn. Foods.

[24] Ali A., Margetts B.M., Zainuddin A.A. (2020). Exploration of the principal component analysis (PCA) approach in synthesizing the diet quality of the Malaysian population. Nutrients.

[25] Musaiger A.O. (1993). Socio-cultural and economic factors affecting food consumption patterns in the Arab countries. J. R. Soc. Health.

[26] Machado P.P., Steele E.M., Levy R.B., da Costa Louzada M.L., Rangan A., Woods J., Gill T., Scrinis G., Monteiro C.A. (2020). Ultra-processed food consumption and obesity in the Australian adult population. Nutr. Diabetes.

[27] Shim J.S., Shim S.Y., Cha H.J., Kim J., Kim H.C. (2021). Socioeconomic characteristics and trends in the consumption of ultra-processed foods in Korea from 2010 to 2018. Nutrients.

[28] Brunner T.A., Van der Horst K., Siegrist M. (2010). Convenience food products. Drivers for consumption. Appetite.

[29] Lam M.C.L., Adams J. (2017). Association between home food preparation skills and behaviour, and consumption of ultra-processed foods: Cross-sectional analysis of the UK National Diet and nutrition survey (2008–2009). Int. J. Behav. Nutr. Phys. Act..

[30] Singapore Department of Statistics (2020). Home Ownership Rate of Resident Households; Unemployment Rate. https://www.singstat.gov.sg/find-data/search-by-theme/households/households/latest-data.

[31] Streeter J.L. (2017). Socioeconomic factors affecting food consumption and nutrition in China: Empirical evidence during the 1989–2009 period. Chin. Econ..

[32] Possa G., Castro M.A.D., Sichieri R., Fisberg R.M., Fisberg M. (2017). Dairy products consumption in Brazil is associated with socioeconomic and demographic factors: Results from the National Dietary Survey 2008–2009. Rev. Nutr..

[33] International Nut and Dried Fruit Council Global Statistical Review 2020–2021. https://www.nutfruit.org/files/tech/1621253983_INC_Statistical_Yearbook_2020-_2021.pdf.

[34] Rehm C.D., Peñalvo J.L., Afshin A., Mozaffarian D. (2016). Dietary intake among US adults, 1999–2012. JAMA.

[35] Siegrist M., Bearth A., Hartmann C. (2022). The impacts of diet-related health consciousness, food disgust, nutrition knowledge, and the Big Five personality traits on perceived risks in the food domain. Food Qual. Prefer..

[36] Monteiro C.A., Cannon G., Levy R., Moubarac J.C., Jaime P., Martins A.P., Canella D., Louzada M., Parra D. (2016). NOVA. The star shines bright. World Nutr..

[37] Bazzano L.A., Serdula M.K., Liu S. (2003). Dietary intake of fruits and vegetables and risk of cardiovascular disease. Curr. Atheroscler. Rep..

[38] Oude Griep L.M., Geleijnse J.M., Kromhout D., Ocké M.C., Verschuren W.M. (2010). Raw and processed fruit and vegetable consumption and 10-year coronary heart disease incidence in a population-based cohort study in the Netherlands. PLoS ONE.

[39] Ruel M.T., Harris J., Cunningham K. (2013). Diet quality in developing countries. Diet Quality.

[40] Hoddinott J., Yisehac Y. (2002). Dietary Diversity as a Food Security Indicator.

[41] Stuckler D., McKee M., Ebrahim S., Basu S. (2012). Manufacturing epidemics: The role of global producers in increased consumption of unhealthy commodities including processed foods, alcohol, and tobacco. PLoS Med..

[42] Jayawardena R., Byrne N.M., Soares M.J., Katulanda P., Yadav B., Hills A.P. (2013). High dietary diversity is associated with obesity in Sri Lankan adults: An evaluation of three dietary scores. BMC Public Health.

[43] Raynor H.A., Epstein L.H. (2001). Dietary variety, energy regulation, and obesity. Psychol. Bull..

[44] Levin K.A. (2006). Study design III: Cross-sectional studies. Evid. Based Dent..

[45] Scarmozzino F., Visioli F. (2020). Covid-19 and the subsequent lockdown modified dietary habits of almost half the population in an Italian sample. Foods.

[46] Sobba W., Landry M.J., Cunanan K.M., Marcone A., Gardner C.D. (2021). Changes in ultra-processed food consumption and lifestyle behaviors following COVID-19 shelter-in-place: A retrospective study. Foods.

[47] Chenarides L., Grebitus C., Lusk J.L., Printezis I. (2021). Food consumption behavior during the COVID-19 pandemic. Agribusiness.

[48] Bonaccio M., Costanzo S., Ruggiero E., Persichillo M., Esposito S., Olivieri M., Di Castelnuovo A., Cerletti C., Donati M.B., De Gaetano G. (2021). Changes in ultra-processed food consumption during the first Italian lockdown following the COVID-19 pandemic and major correlates: Results from two population-based cohorts. Public Health Nutr..

[49] Crino M., Barakat T., Trevena H., Neal B. (2017). Systematic review and comparison of classification frameworks describing the degree of food processing. Nutr. Food Technol..

[50] Eicher-Miller H.A., Fulgoni III V.L., Keast D.R. (2012). Contributions of processed foods to dietary intake in the US from 2003–2008: A report of the Food and Nutrition Science Solutions Joint Task Force of the Academy of Nutrition and Dietetics, American Society for Nutrition, Institute of Food Technologists, and International Food Information Council. J. Nutr..

[51] Monteiro C.A., Levy R.B., Claro R.M., Castro I.R.R.D., Cannon G. (2010). A new classification of foods based on the extent and purpose of their processing. Cad. Saude Publica.

[52] Asfaw A. (2011). Does consumption of processed foods explain disparities in the body weight of individuals? The case of Guatemala. Health Econ..

[53] Poti J.M., Mendez M.A., Ng S.W., Popkin B.M. (2015). Is the degree of food processing and convenience linked with the nutritional quality of foods purchased by US households?. Am. J. Clin. Nutr..

[54] Martinez-Perez C., San-Cristobal R., Guallar-Castillon P., Martínez-González M.Á., Salas-Salvadó J., Corella D., Castañer O., Martinez J.A., Alonso-Gómez Á.M., Wärnberg J. (2021). Use of different food classification systems to assess the association between ultra-processed food consumption and cardiometabolic health in an elderly population with metabolic syndrome (PREDIMED-Plus Cohort). Nutrients.

[55] Braesco V., Souchon I., Sauvant P., Haurogné T., Maillot M., Féart C., Darmon N. (2022). Ultra-processed foods: How functional is the NOVA system?. Eur. J. Clin. Nutr..

[56] Petrus R.R., do Amaral Sobral P.J., Tadini C.C., Gonçalves C.B. (2021). The NOVA classification system: A critical perspective in food science. Trends Food Sci. Technol..

[57] Willett, Walter (2012). Nutritional Epidemiology.

