# Supplementary material for: Consumption Patterns of Processed Foods in Singapore—A Cross-Sectional Study
Source: Foods. 2022 Sep 9;11(18):2782. doi: 10.3390/foods11182782 (PMC9498269; doi:10.3390/foods11182782)
Supplement: Supplementary file 1 [file foods-11-02782-s001.zip › foods-1831291-supplementary.pdf]

foods MDPI

Supplementary Information

Table S1. Food items in the processed food groups

Processed food group	Processed food item
Beverages, excluding dairy products	Bubble tea<br>Cereal beverage<br>Soya bean drink
Cereals and cereal products	Bean thread noodles (tang hoon)<br>Buckwheat noodles (soba, naengmyeon)<br>Cereal & Grain flour<br>Cereal Groats And Meal<br>Coconut Flour<br>Dried Rice Cake<br>Dry instant noodles<br>Egg Tofu<br>Gluten<br>Glutinous Rice Flour<br>Instant noodles with soup<br>Maize Flour<br>Maize starch<br>Mock Meat<br>Oat cracker snacks<br>Other cereal based snack Unspecified<br>Other Flour Unspecified<br>Other noodles Unspecified<br>Other rice products Unspecified<br>Other starch Unspecified<br>Plant and Bean Based Meal Cereal<br>Popcorn<br>Potato starch<br>Processed cereal grain<br>Rice dumpling<br>Rice Flour<br>Rice noodles (pho noodles, hor fun)<br>Rice puff<br>Roasted Malt<br>Root and tuber flour<br>Tapioca starch<br>Vermicelli<br>Wheat noodles (udon, somen, la mian)<br>Wheat starch<br>Yellow and egg noodles (ramen, you mian)
Confectionery and sweeteners	Cocoa Paste<br>Cocoa Powder<br>Fruit jelly<br>Hard candy (including lollipop)<br>Honey<br>Instant Pudding with and without Fruits (Jell-O)<br>Nougat<br>Preserved Nata De Coco

	Sugar Syrup
Dairy products	Dairy Cream<br>Buttermilk<br>Fermented milk drink<br>Non-Dairy Cream<br>Yogurt
Fish and seafood	Canned bivalvia (Shell fish)<br>Canned cephalopods (Squid, octopus, cuttlefish)<br>Canned crab<br>Canned fish<br>Canned lobster<br>Canned prawn/shrimp<br>Crab balls<br>Crab cake<br>Crab nuggets<br>Crab paste<br>Crab Pate/burger<br>Dried Fish<br>Fish balls<br>Fish cake<br>Fish floss<br>Fish nuggets<br>Fish paste<br>Fish pate/burger<br>Fish sausage/ hotdog / cocktail<br>Imitation abalone<br>Imitation crabstick<br>Lobster balls<br>Lobster cake<br>Lobster paste<br>Lobster pate/burger<br>Other dried seafood and other related products Unspecified<br>Other dried seafood snack Unspecified<br>Other imitation Seafood Unspecified<br>Other salted seafood and related products Unspecified<br>Other seafood cake and related products Unspecified<br>Other seafood canned and related products Unspecified<br>Other seafood preserved and related products Unspecified<br>Other seafood smoked and related products Unspecified<br>Prawn/shrimp balls<br>Prawn/shrimp cake<br>Prawn/shrimp nuggets<br>Prawn/shrimp paste<br>Prawn/shrimp Pate/burger<br>Preserved bivalvia (Shell fish)<br>Preserved cephalopods (Squid, octopus, cuttlefish)<br>Preserved crab<br>Preserved echinoderms (Sea urchins, sea cucumber)<br>Preserved fish<br>Preserved prawn/shrimp<br>Salted fish<br>Smoked bivalvia (Shell fish)<br>Smoked cephalopods (Squid, octopus, cuttlefish)

	Smoked crab
	Smoked echinoderms (Sea urchins, sea cucumber)
	Smoked fish
	Smoked gastropods (Escargot)
	Smoked lobster
	Smoked prawn/shrimp
	Squid balls
	Squid paste
Fruits	Apple Paste and puree
	Apricot Paste
	Citrus Fruit Paste
	Coconut Milk
	Coconut Paste and puree
	Dried banana
	Dried berries
	Dried tangerine
	Fruit jam/marmalade
	Fruit Pastes and puree with Mixed Fruit
	Fruit Pastes Berry Fruit
	Orange Puree
	Other Coconut products Unspecified
	Other dried fruit Unspecified
	Other fruit paste and puree Unspecified
	Other pickled fruit Unspecified
	Other Preserved Fruit Unspecified
	Other salted fruit Unspecified
	Other sugared fruit Unspecified
	Pickled Apple
	Pickled Mixed Fruit
	Pickled Tamarind
	Plum Paste
	Salted Olive
	Salted Plum
	Salted Prune
	Sugared Apricot
	Sugared Arbutus (E.g. Yang mei)
	Sugared Banana
	Sugared berries
	Sugared Citrus Peel
	Sugared Tangerine
Meat and meat products	Beef Bacon
	Beef Ham and Bologna
	Beef Luncheon Meat
	Beef Meat ball
	Beef Pate/Burger
	Beef Satay
	Beef Sausage/hot dog/cocktail
	Canned Beef
	Canned Chicken
	Canned Duck
	Canned Mutton
	Canned Pork

	Chicken Bacon
	Chicken Ham and Bologna
	Chicken Luncheon Meat
	Chicken Meat ball
	Chicken Nuggets
	Chicken Pate/Burger
	Chicken Satay
	Chicken Sausage/hot dog/cocktail
	Cured Chicken
	Duck Ham and Bologna
	Duck Sausage/hot dog/cocktail
	Meat floss
	Mutton Pate/Burger
	Mutton Satay
	Pork Bacon
	Pork Ham and Bologna
	Pork Luncheon Meat
	Pork Meat ball
	Pork Pate/Burger
	Pork Satay
	Pork Sausage/hot dog/cocktail
	Processed exotic animal product
	Smoked Beef
	Smoked Chicken
	Smoked Duck
	Smoked Pork
	Smoked Turkey
	Turkey Ham and Bologna
	Turkey Sausage/hot dog/cocktail
Nuts and seeds	Dried Tree Nuts
	Nut and seed puree and spread
	Other Tree Nuts Unspecified
	Preserved Tree Nuts
	Roasted Nuts Snack
	Sugared Tree Nuts
Sauces and condiments	Barbeque Sauce
	Honey mustard
	Mayonnaise
	Other Dipping Sauces Unspecified does not include tomato
	Thousand island sauce
Seaweed and fungi	Dried Jew Ear
	Dried Mushroom
	Dried Seaweed
	Other Fungi Unspecified
	Other Seaweed Unspecified
	Pickled Mushroom
	Processed Seaweed
Soups	Canned Soup/Broth
	Instant Soup Powder
	Meat Extract Broth/soup
	Packet broth and soup
Vegetables	Dried bean

	Dried Honey Tomato (not cherry tomato)
	Other dried vegetable Unspecified
	Other pickled vegetable Unspecified
	Other salted vegetables Unspecified
	Other sugared vegetables Unspecified
	Other vegetable puree Unspecified
	Pickled Asparagus
	Pickled Bamboo shoot
	Pickled bean
	Pickled Ginger
	Pickled Mixed Vegetable
	Pickled Onion
	Pickled tomato
	Salted beans vegetables (black bean)
	Salted Cabbage
	Salted Potato
	Salted Sweet Corn
	Sugared Gourds And Melon (melon peel)
	Sugared sweet corn
	Tomato Puree
